# Measurement of the point spread function of a pixelated detector array

**DOI:** 10.1186/2197-7364-2-S1-A10

**Published:** 2015-05-18

**Authors:** Christian Ritzer, Patrick Hallen, David Schug, Volkmar Schulz

**Affiliations:** Department of Physics of Molecular Imaging Systems, Institute for Experimental Molecular Imaging, RWTH Aachen University, Aachen, Germany

In order to further understand the PET/MRI scanner of our group, we measured the point spread function of a preclinical scintillation crystal array with a pitch of 1 mm and a total size of 30 mm ~ 30 mm ~ 12 mm. It is coupled via a lightguide to a dSiPM from Philips Digital Photon Counting, used on the TEK-setup. Crystal identification is done with a centre of gravity algorithm and the whole data analysis is performed with the same processing software as for the PET insert, giving comparable results. The beam is created with a 22 NA-Point-Source and a lead collimator, with 0.5 mm bore diameter. The algorithm sorted 62 % of the coincidences into the correct crystal.

